# Investigating awareness of artificial intelligence in healthcare among medical students and professionals in Pakistan: a cross-sectional study

**DOI:** 10.1097/MS9.0000000000001957

**Published:** 2024-03-18

**Authors:** Mohammad Umer, Aiman Naveed, Qanita Maryam, Arif Rasheed Malik, Naghmana Bashir, Kamal Kandel

**Affiliations:** a King Edward Medical University (KEMU); b Fatima Jinnah Medical University, Lahore, Pakistan; c Kathmandu University, Dhulikhel, Nepal

**Keywords:** artificial intelligence, big data, deep learning, machine learning, precision medicine

## Abstract

**Objective:**

The purpose of this study is to find out the level of awareness and acceptance of artificial intelligence (AI) in Pakistan’s medical community so as to comment on its future in our healthcare system.

**Methods:**

A survey consisting of 15 close-ended questions was conducted. The questions inquired about awareness about AI and discovered the opinions of healthcare professionals regarding its benefits and expected problems. The data were analyzed using SPSS version 26, and descriptive statistics for percentage and frequency were computed. χ^2^ test was used to analyze the subgroups (Significant *p* value <0.05).

**Results:**

A total of 351 participants were included in this study. General familiarity with AI was low. Only 75 (21.3%) participants answered that they had good familiarity with AI, and only 56 (16%) of them had good familiarity with the role of AI in medicine. One hundred sixty-eight (47.9%) participants disagreed that AI would out-compete the physician in the important traits of professionalism. Only 71 (20.2%) participants believed AI to be diagnostically superior to the physician. Two hundred fourteen (61.0%) were worried about completely trusting AI in its decisions, and 204(58.1%) believed that AI systems lacking human traits would not be able to mirror the doctor-patient relationship. Two hundred sixty-one (74.4%) participants believed that AI would be useful in Administrative tasks. A majority, 162 (46.2%), do not believe that AI would replace them. Finally, a huge majority of participants [225 (64.1%)] demanded the integration of AI in Pakistan’s healthcare system.

**Conclusion:**

This study suggests that a majority of healthcare professionals in Pakistan do not believe that they are sufficiently aware of the role of AI in healthcare. This was corroborated by their answers to various questions regarding the capabilities of AI. This study indicates the need for a more comprehensive ascertainment of healthcare professionals’ perceptions regarding the role of Artificial Intelligence in medicine and bridging the gap between doctors and technology to further promote a patient-centred approach to medicine.

## Introduction

Highlights

- A total of 351 participants completed a survey with 15 close-ended questions about their awareness and opinions of artificial intelligence (AI) in healthcare.
- Results showed that general familiarity with AI was low, with only 21.3% of participants reporting good familiarity.
- While many participants were worried about completely trusting AI in its decisions and believed that AI lacked human traits, a majority (74.4%) believed that AI would be useful in administrative tasks.
- These findings suggest that there is support for the integration of AI in Pakistan’s healthcare system, but also highlight concerns about trust and human-like qualities.

Artificial intelligence generally refers to using computers to mimic or imitate human intelligence without human intervention^1^. This growing technology, with its impressive arsenal of learning algorithms and decision-making abilities, has found its way into nearly all major aspects of life including the field of medicine. AI is currently augmenting the field of medicine in diagnosis, administration, disease prediction and treatment strategies^2^ in many parts of the world.

For example, AI can be used to aid in diagnosis by interpreting radiology scans and integrating multiple data systems, that is radiology and pathology, to make diagnosis^3^. It can be used to split mammograms^4^ and retinal fundus images^5^ into pixel-level variables and detect anomalies at an early stage that previously went undetected^4^. AI devices developed by accuray, in particular, have the ability to track and attack tumours^6^. For surgery, AI bots like DaVinci or those developed by VicariousSurgical have enabled high-precision, minimally invasive surgeries^7^. Moreover, a company called Enlitic has developed AI systems that utilize patients’ history, serology and radiology scans, ECG, EKG, genomics data to diagnose pathologies of different systems in the body^8^. Hospital administration, patient data compilation and processing, patient profiling and referral^9^ and maintaining databases are tasks that can be made easier^8^ by means of AI-based systems such as those developed by Nuance^10^. AI tools like Electronic health records (EHR)^11^ and concepts like BigData enable the collection and analysis of patient data for diagnosis and research purposes.

However, AI comes with its own set of challenges that range from a possible risk of increased unemployment to concerns about patient privacy, trust and morality^12,13^ and the lack of explainability in their approach to problem-solving^14^.

The above-mentioned merits and demerits of AI lead us to question the current position of AI in the healthcare system, as well as its potential in the future. More specifically, in a country like Pakistan this question becomes more important since the country’s healthcare system is also fighting the uphill battle of keeping up with the financial demands of healthcare^15^ an area where AI-assisted tools are expected to be of help^16^.

Irrespective of the debate that weighs the potential and the problems of AI against each other, we believe that the biggest stakeholders that would decide the future of AI in medicine are the healthcare professionals. It’s imperative that we discover their level of awareness and their stance on these new developments. Therefore, this study aims to explore and weigh the opinions of the medical community of Pakistan regarding AI’s future in our healthcare system and its positive and negative impacts.

## Materials and methods

### Ethical review statement

The proposal was discussed in the Institutional Review Board of the parent institute and was unanimously approved in its meeting.

### Study design

We conducted a cross-sectional study to assess the awareness of AI among medical students and practitioners. A survey questionnaire was designed using prior questionnaires that assess awareness about AI among medical students and doctors.

### Selection and description of participants

Participants were selected on the basis of the inclusion/exclusion mentioned below. They were categorized into medical students, training physicians and medical practitioners.

### Study setting

Google Forms (online platform). The study was conducted in multiple centres. Questionnaire was distributed to multiple medical colleges/hospitals in Punjab (province) and Karachi (city).

### Study duration

Three months.

### Sampling method

A snowball sampling technique was used to recruit the participants. Google form questionnaire was distributed online through emails, text messages, Whatsapp status, and friends’ circles.

### Sample size calculation with reference

The sample size of 213 participants was calculated by taking the confidence level as 95%, absolute precision as 5%, and the expected percentage of participants finding AI useful as 83.4%, based on previous surveys^17^.

### Inclusion and exclusion criteria

Undergraduate Medical Students, House Officers, Post Graduate Residents and Practitioners currently studying or working in Pakistan who can read and understand English and have access to the internet were included in this study.

Nonresidents of Pakistan, people belonging to other professions, those who cannot read and understand English, those who did not have access to the internet or those who did not consent to be a part of this study were excluded from it.

### Statistical analysis

Statistical analysis was performed using IBM SPSS version 26. Descriptive statistics for frequency and percentage were acquired for all variables. Categorical variables like gender and working status are presented as frequency and percentage. Continuous variables are presented as mean ± SD. In subgroup analysis, χ^2^ test was used to compute results for the association of age, sex and working status with differences in responses to all questions. A *p* value of less than 0.05 was taken to be significant.

We investigated whether the responses of the participants differed from each other according to their age, sex and working status. The categories for working status were medical student, training physician and medical practitioner. The purpose of such subgrouping was to ascertain if the accruing experience in training physicians and medical practitioners, compared to the students, presented a difference of opinions between the subgroups.

The work has been reported as being in line with the STROCSS criteria.^16^

## Results

### Participants

Written consent was taken from the participants at the beginning of the questionnaire.

#### Demographics

A total of 351 people participated in this research. Two hundred five (58.4%) were females and 146 (41.6%) were males. The demographic characteristics are summarized in Table 1.

**Table 1:** Age, gender and working status of participants.

		*n* (%)	
Variable	Responses	Male	Female
Age	15–25	126 (35.9)	164 (46.7)
	25–35	16 (4.6)	28 (8.0)
	35–45	3 (0.9)	6 (1.7)
	45–55	0	3 (0.9)
	55–65	0	2 (0.6)
	>65	1 (0.3)	2 (0.6)
Working status	Medical Student	121 (34.4)	165 (47.0)
	Training physician	18 (5.1)	26 (7.4)
	Medical practitioner	7 (1.9)	14 (3.98)
Total		146 (41.6)	205 (58.4)

#### Working status

Of the 351 participants, 286 (81.5%) were medical students, 44 (12.5%) were training physicians and 21 (6.0%) were medical practitioners. The professional characteristics of the participants are listed in Table 1.

### Questionnaire

The responses to the various questions are summarized in Table 2.

**Table 2:** Familiarity with artificial intelligence and its role in medicine.

	Yes	No	Maybe	Total
Questions	*n* (%)	*n* (%)	*n* (%)	*n* (%)
Do you think AI systems would be superior to the traditional physicians in making diagnosis and prescribing curative treatments?	71 (20.2)	117 (33.3)	163 (46.4)	351 (100.0)
Do you think administrative tasks like patients’ data management would be improved if managed by AI-powered soft wares?	261 (74.4)	14 (4.0)	76 (21.7)	351 (100.0)
Are you particularly worried about completely trusting AI algorithms with patients’ lives?	214 (61.0)	56 (16.0)	81 (23.1)	351 (100.0)
Do you think AI systems lacking human traits like empathy and compassion can exactly mirror the traditional doctor-patient relationship?	99 (28.2)	204 (58.1)	48 (13.7)	351 (100.0)
Do you think AI systems would have the professional attitude to work autonomously in the Healthcare system?	127 (36.2)	131 (37.3)	93 (26.5)	351 (100.0)
Do you think that AI systems would possess the necessary knowledge to perform efficiently in the healthcare system?	159 (45.3)	64 (18.2)	128 (36.5)	351 (100.0)
Do you think AI systems would display the necessary skills required to perform its tasks accurately in the Healthcare system?	146 (41.6)	63 (17.9)	142 (40.5)	351 (100.0)
Do you think that AI systems would out-compete physicians in the aforementioned parameters of professionalism that is attitude, knowledge and skill?	61 (17.4)	168 (47.9)	122 (34.8)	351 (100.0)
Do you as a practicing doctor or future aspirant fear that your job would be at stake due to introduction of AI in medicine?	76 (21.7)	162 (46.2)	113 (32.2)	351 (100.0)
Do you think students should be made to attend courses regarding management of AI systems in addition to traditional clinical studies?	257 (73.2)	31 (8.8)	63 (17.9)	351 (100.0)
Do you think inclusion of AI can solve the current problems faced by the healthcare system (low doctor-patient ratio, lack of efficiency and accuracy, financial burden)?	167 (47.6)	45 (12.8)	139 (39.6)	351 (100.0)
Do you demand the Integration of AI technology to improve the healthcare system in Pakistan?	225 (64.1)	37 (10.5)	89 (25.4)	351 (100.0)

AI, artificial intelligence.

#### Responses to the questionnaire

General familiarity with AI among participants was average [Mean ± SD; 2.63±1.07]. As for the familiarity with the role of AI in medicine, only 56(16%) of the participants answered that they were considerably familiar with the role of Artificial Intelligence in Medicine [Mean ± SD; 2.40 ± 1.06].

Responses to questions about the attitude of participants towards AI in the healthcare system showed varied results.

Most of the participants, 261 (74.4%), believed that AI-powered software could improve the management of administrative tasks in the healthcare system.

Most of the participants 214 (61.0%) answered that they would not completely trust AI algorithms with patients’ life.

When asked if AI-powered systems would essentially out-compete physicians’ attitude, knowledge and skills, a majority [168 (47.9%)] of participants disagreed. The majority of participants [162 (46.2%)] answered that they do not fear that they’ll risk losing their jobs if AI-based systems were integrated into the health care systems.

Regarding the potential of AI in health, 225 participants demanded its integration into the healthcare system of Pakistan.

**Figure 1:**
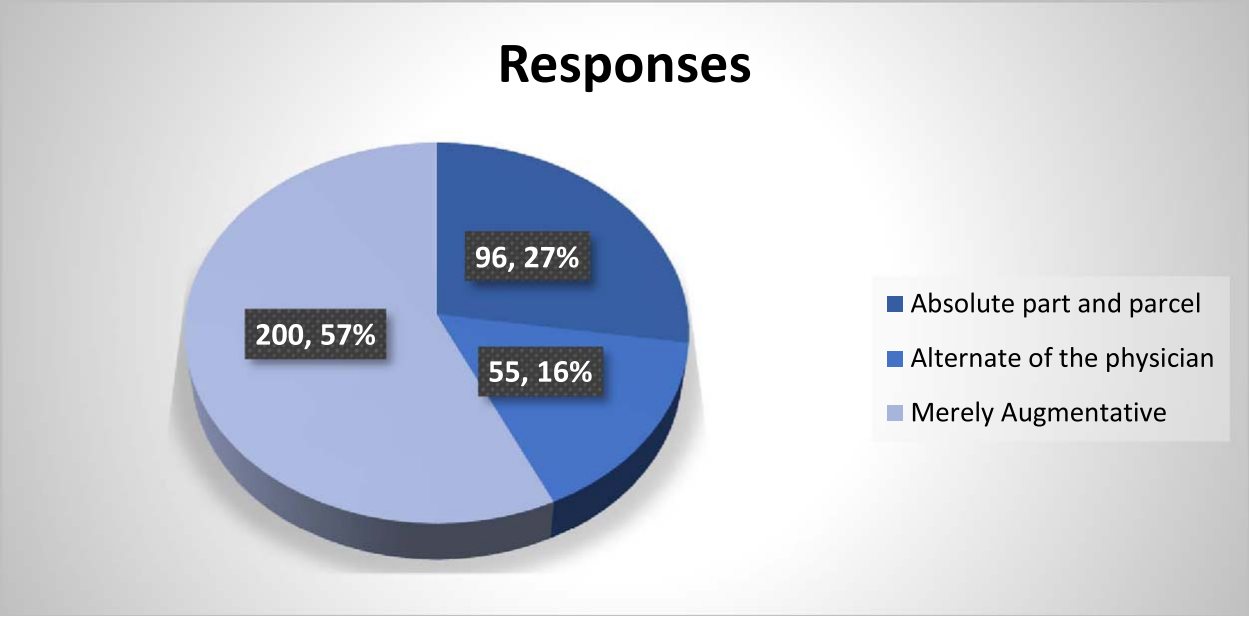
Specific role of artificial intelligence in healthcare.

Responses to the question about the specific role of AI in medicine are summarized in Fig. 1.

### Subgroup analysis

Only those differences in responses were considered to be statistically significant and had a *p* value of less than 0.05 according to the χ^2^ test.

#### Age and sex

There was no significant difference in responses according to age and sex.

#### Working status

There were significant differences in responses according to the working status for four questions (Q7, Q8, Q11, Q14). The results are summarized in Table 3.

**Table 3:** Subgroup analysis.

		Working status			
Question	Responses	Medical student, *n* (%)	Training physicians, *n* (%)	Medical practitioner, *n* (%)	*P*
Would AI possess the professional attitude?	Yes	98 (34.3)	22 (50.0)	7 (33.3)	0.038
	No	103 (36.0)	18 (40.9)	10 (47.6)	
	Maybe	85 (29.7)	4 (9.1)	4 (19.0)	
Would AI possess the necessary knowledge?	Yes	124 (43.4)	24 (54.5)	11 (52.4)	
	No	46 (16.1)	13 (29.5)	5 (23.8)	0.012
	Maybe	116 (40.6)	7 (15.9)	5 (23.8)	
Do you think inclusion of AI can solve the current problems faced by the healthcare system?	Yes	128 (44.8)	25 (56.8)	14 (66.7)	
	No	34 (11.9)	9 (20.5)	2 (9.5)	0.029
	Maybe	124 (43.4)	10 (22.7)	5 (23.8)	
What would be the specific role of AI in healthcare?	Absolute part and parcel	80 (28.0)	13 (29.5)	3 (14.3)	
	Alternate of the physician	36 (12.6)	14 (31.8)	5 (23.8)	0.006
	Merely augmentative	170 (59.4)	17 (38.6)	13 (61.9)	

AI, artificial intelligence.

## Discussion

The idea of AI integration into everyday healthcare practice is still in the initial phase in Pakistan. Currently, the use of AI in public healthcare is limited to the area of telemedicine^18^. The National Center of Artificial Intelligence (NCAI) is a collaboration between the Law and Justice Commission of Pakistan and the National University of Science and Technology (NUST) that promotes AI-based healthcare innovation in a limited capacity^18,19^. Generally, however, exposure to AI use in clinical settings is not very common among medical practitioners in this country^20^. Therefore, our aim was to gauge the current understanding and familiarity with AI in Pakistan’s medical community with this context in full view.

The familiarity and awareness about artificial intelligence and its role in medicine were generally low among medical students and physicians. However, most of the participants did believe that the inclusion of AI-based programs would significantly decrease the problems of the healthcare system like lack of efficiency, low doctor-to-patient ratio, and immense financial burden. The majority of the participants believed that AI-based programs could efficiently perform administrative tasks like patient data management. They also believed that physicians are superior in making diagnoses and that the best role for AI would be to augment the duties of the physician. This displays a belief in the capabilities of what AI could do even though they lack knowledge and understanding of AI and have little exposure to its practical implementation.

This is consistent with prior research. A study conducted in 2022 concluded that participants displayed low familiarity with AI. Only 35% knew about Machine learning and Deep Learning, but still, a majority of medical students (69.6%) and doctors (81.8%) believed that AI could be of considerable assistance as a physician’s aid in diagnosis and management^20^. Another study conducted in UK found that about three-fourths of medical students believe the role of AI to be essential in medicine and healthcare^21^.

The majority of the participants in the survey agreed that adding information about AI’s applicability to the medical curriculum would be a good idea. A prior study is the US found similar results where most of the study participants thought that the addition of AI literacy to medical education would be beneficial^22^.

Most of the participants of this survey believe that the integration of AI in the healthcare systems would not put their jobs at risk. This is consistent with a prior survey held in 2019 that showed that doctors are less likely to be worried about AI replacing their jobs^17^. However, a 2017 research showed that generally, most people (72% of the 4135 participants) express worry that their jobs would be at risk in a future where robots and computers can do many human jobs^23^.

A majority of the participants also believed that AI systems could not exactly mirror traditional doctor-patient relationships owing to the lack of ability to show empathy and compassion towards patients. Additionally, participants displayed a certain amount of mistrust for AI’s decision-making abilities. Our findings mirror that of a prior study in Turkey where 45.5% of participants thought that the use of AI in medicine would damage trust, and 42.7% thought that it would negatively impact the doctor-patient relationship^24^.

At this point, it is important to mention some concerns regarding the applicability of AI in medicine. Firstly, there is the question about how effectively AI-derived knowledge can be applied universally, which may limit its diagnostic accuracy across diverse populations^4^. Another concern with certain AI models is their lack of transparency in decision-making, which affects trust and confidence among medical professionals^14,25^. The use of Big Data in AI systems also raises worries about breaches in patient confidentiality and data security^26^. While there are fears that AI may replace physicians, integrating AI technology courses into medical school curricula can help ensure that it complements rather than replaces human expertise^27^.

Results of subgroup analysis showed that physicians differed from medical students at some points. Medical students gave the answer “maybe” far more frequently when questioned about the knowledge and attitude of AI as well as its potential to solve some of the biggest problems currently faced by the healthcare system in Pakistan. Compared to this, physicians gave the decisive answers “yes” or “no” more frequently. This difference was found to be significant statistically, and the reason for this could be the greater experience and knowledge of the practical aspect of healthcare they possess compared to medical students. Moreover, more physicians believe that AI would be an alternative to the physician, which could be attributed to a number of things, including greater experience and more exposure to AI in practice.

There were a few limitations to our study. Firstly, the number of people that participated was quite low. Secondly, the ratio of practicing doctors to participants in our study was too low. The reason for this disproportion is the sampling technique we used. The snowball sampling technique is a form of non-probability sampling that recruits new participants through already recruited participants^28^. Since data collection was undertaken majorly by undergraduate medical students, it is easy to understand why the majority of participants were recruited from among medical students. This limitation makes it important to interpret the results of our study with caution, especially regarding the difference in opinions among medical students and professionals in response to some of the survey questions. Thirdly, this research was not conducted by AI experts. Finally, patients, who need to have just as big a say in the future of our healthcare system, were not included in this research. However, this limitation is inherent to the objective and design of this study as it aimed to gauge the awareness about AI in the medical community of Pakistan and not among the general population or patients.

The implications of our study are two-fold. First, it can be considered a contribution to the current understanding of AI and its use in healthcare in the medical community, especially medical students. This can help policymakers gauge the readiness of the medical community of Pakistan to accept AI and work towards developing an overall more informed, efficient and equitable healthcare system for all. Secondly, as our study suggests the readiness of the participants to accept additions to medical education that will help them better understand AI, policies to enact this possibility of making medical students “AI literate” can be considered. Moreover, our study indicates the need to further investigate the concerns of medical professionals regarding job security and trust through country-wide qualitative and quantitative surveys and research.

## Conclusion

Our study found that AI is still an unfamiliar topic for many medical students and professionals in Pakistan. However, the demand for integration of AI in Pakistan’s healthcare system shows that despite the lack of awareness and various limitations of AI, the medical community of Pakistan still believes that the solution to many problems of our healthcare system lies with the proper use of AI. This study also indicates the need for a more comprehensive ascertainment of healthcare professionals’ perceptions regarding the role of Artificial Intelligence in medicine and bridging the gap between doctors and technology to further promote a patient-centred approach to medicine.

## Ethical approval

Ethical approval for this study (Institutional Review Board (IRB), King Edward Medical University protocol no. 648/RC/KEMU) was provided by the Ethical Committee of IRB King Edward Medical University, Neela Gumbad, Anarkali Lahore, Pakistan on 05 September 2020.

## Consent

Written informed consent was obtained from the participants for publication and any accompanying images. A copy of the written consent is available for review by the Editor-in-Chief of this journal on request.

## Source of funding

None to declare.

## Author contribution

M.U.: methodology, formal analysis, resources, writing—original draft preparation. A.N.: conceptualization, data curation, formal analysis, writing—original draft preparation. Q.M.: formal analysis, data curation, writing—original draft preparation. A.R.M.: visualization, writing—review and editing, project administration. N.B.: resources, visualization, writing—review and editing. K.K.: conceptualization, review and editing, project administration.

## Conflicts of interest disclosure

There was no conflict of interest between authors.

## Research registration unique identifying number (UIN)

There were no human participants of this research. Research has been registered on Open Science Framework registry. Unique identifying Number: https://doi.org/10.17605/OSF.IO/ET6KR.

## Guarantor

Mohammad Umer.

## Data availability statement

All data are available upon reasonable request

## Provenance and peer review

Not commissioned, externally peer-reviewed.
